# “Pay Attention! Pay Attention! Pay Attention!!!”: The Pivotal Role of Educators and the Educational System as Experienced by Survivors of Child Sexual Abuse

**DOI:** 10.3390/bs14050419

**Published:** 2024-05-16

**Authors:** Laura I. Sigad, Dafna Tener, Efrat Lusky-Weisrose, Jordan Shaibe, Carmit Katz

**Affiliations:** 1 Department of Inclusive Education, Faculty of Graduate Studies, Oranim College of Education, Kiryat Tiv’on 3600600, Israel; jordan.shaibe@mail.huji.ac.il; 2 The Paul Baerwald School of Social Work and Social Welfare, Mount Scopus Campus, Hebrew University of Jerusalem, Jerusalem 9190500, Israel; dafna.tener@mail.huji.ac.il (D.T.); efratlusky@gmail.com (E.L.-W.); 3 Bob Shappell School of Social Work, Tel Aviv University, Tel Aviv 6997801, Israel; carmitkatz@tauex.tau.ac.il

**Keywords:** child sexual abuse (CSA), child sexual abuse disclosure, educators, educational system, testimonies, help seeking behaviors

## Abstract

Educational institutions and educators are significant in children’s lives, and they have a crucial role in implementing policies, practices, and sexual education to enhance children’s safety. Such policies and practices should be based on the voices of CSA survivors. This study explored child sexual abuse (CSA) survivors’ viewpoints on their past experiences with educators and the educational system. A qualitative thematic approach was used to analyze 61 written testimonies collected in 2020–2021 by the Israeli Independent Public Inquiry on CSA. Two interrelated themes arose: (1) CSA survivors’ retrospective perspectives of educators and the educational system’s responses to signs of their CSA, described as ranging from abusive to life-saving. Specifically, they shared three types of responses: (a) harmful and hurtful; (b) dismissive and ignoring; and (c) accepting and attending. (2) The second theme described the survivors’ messages to educators to promote constructive change. The survivors conveyed expectations that educators should play a central role in CSA prevention, detection, and intervention and, specifically, the need for educators to receive professional training, provide beneficial sexual education, and identify and respond to CSA. The findings promoted moving beyond individual-level interventions to focus on improving educational institutional and organizational cultures related to CSA in both national and international contexts.

## 1. Introduction

Educators are known to play a key role as recipients of child sexual abuse (CSA) disclosures and in addressing and supporting their students in navigating the negative consequences of the abuse [1,2]. To better understand and inform educators and the educational systems’ responses to CSA, it is crucial to listen and learn from CSA survivors who hold valuable lived experience. Although there are often national and local laws and guidelines in place to address CSA, educational systems are frequently involved in enforcing them due to their regular contact with students. Therefore, it is imperative for educators and educational institutions to create an environment of safety and trust to further encourage children to disclose abuse [3]. However, due to the lack of knowledge and training available to educators, they are often left not knowing how to effectively respond to CSA [4,5], which can result in educators taking the actions they see fit [6] rather than acting on knowledge-based policies. Taken together, CSA training alongside engendering an environment of trust and safety should inform each educational institution’s approach to CSA cases [7].

Despite the importance of educators and the educational system in addressing CSA, there is a research gap regarding the responses of the educational system and educators to the disclosure and help-seeking behaviors of CSA survivors. Moreover, there is a lack of survivors’ perspectives about the support and responses they receive. Thus, the current study aimed to understand how survivors experience and perceive the responses of the educational system and their past educators to CSA.

This study adopts Mathews and Collin-Vézina’s [8] definition of CSA, which states that CSA includes sexual acts with direct physical and/or indirect contact involving a child under the age of 18 who is, therefore, unable or has a limited ability to consent. Furthermore, this definition acknowledges that children are vulnerable due to the power dynamic between the person who committed the CSA and the child. In the current study, the term CSA is used in reference to various types of CSA occurring inside and outside the educational setting (e.g., intrafamilial CSA and CSA by a stranger) [9,10]. 

The existing research has pointed to two main groups within the educational system: school employees who commit sexual abuse, including educators (e.g., [11,12]), and peers who engage in problematic and/or harmful sexual behavior (PSB; e.g., [13,14]). School employee sexual misconduct is defined as “any conduct directed toward a student which creates a sexually hostile learning or school environment,” such as sexual comments and advances, exposure to sexual content, and sexual violence ([11], p. 105). PSB, the other major form of abuse found to occur in educational institutions, overlaps with other categories of violence that children may experience, such as violence between adolescent partners, sexual harm and manipulation, bullying and harassment, and drug-related offenses [15]. It is important to recognize that while PSB should be differentiated from CSA committed by adults, children who have experienced PSB may experience this distinction as harmful in itself. As such, the focus should be on responses to the behavior rather than labels, emphasizing rehabilitation over retribution in addressing PSB during this unique developmental period [16,17]. Moreover, the disclosure and reporting of PSB and CSA to school employees are characterized by various barriers, including normalization of the abuse and inadequate responses from the school system [13,18].

Studies conducted among CSA survivors and traumatized children regarding their experiences with the education system have primarily focused on how the abuse might have impacted their educational performance [15,19,20] and how to optimize their learning environment [21]. However, the literature addressing perceptions of the educational system’s role following exposure to students’ sexual victimization is still limited. Furthermore, the literature has revealed disparities between the potential of educators to protect children, the laws and procedures they are obligated to uphold, and their abilities to meet these expectations. When this gap has been explored, it was primarily from educators’ viewpoints (e.g., [22]), while the perspectives of CSA survivors regarding the educators’ roles and responses are notably missing.

As educational institutions and educators are critical to the development and well-being of children and youth, they have a crucial role in implementing policies, practices, and sexual education to enhance children’s safety. Such policies and practices should be based on the voices of CSA survivors themselves. Consequently, survivors of CSA should be considered “experts by experience” [23] and be provided with a platform to generate the knowledge and desired solutions for their recovery and to assist others in similar situations. The present study explored the viewpoints of CSA survivors’ regarding their past experiences with educators and the educational system, guided by the following research questions: (1) How do adults who were sexually abused as children retrospectively describe the ways in which they sought help from within the educational system? (2) How did adult CSA survivors experience educational institutions and educators’ responses following their CSA disclosures? (3) How do adult CSA survivors perceive the roles and responsibilities of the educational system and educators in the context of CSA prevention, identification, and treatment?

### 1.1. CSA Survivors’ Help-Seeking Behaviors

Help-seeking behaviors are defined as searching for and using formal or informal assistance in addressing concerns or challenges [24]. Such behaviors include seeking advice and assistance from others to understand the problem, receiving information and treatment, and obtaining general support [25]. For example, a help-seeking behavior may be disclosing CSA to formal authorities, such as the police, or informal sources, such as family or friends [24]. In general, children’s help-seeking behaviors are influenced by a variety of factors. For example, in the case of eating disorders, younger age has been found to negatively influence help-seeking behaviors and be related to lower mental health literacy, such as holding beliefs that emphasize the ability to recover independently without assistance [26]. Another factor found to influence help-seeking behaviors was gender. In the context of a sexual assault hotline, gender was found to influence both children’s and adult’s help-seeking behaviors. Girls and women had longer calls and sought help from the hotline in addition to support from professionals, friends, and relatives, while boys and men had shorter calls and sought help from the hotline due to a lack of support in their surroundings [27]. Finally, ethnic and cultural contexts were also found to be influential in help-seeking behaviors. For example, while more liberal gender and sexuality beliefs were generally correlated with informal help-seeking behaviors following CSA, among non-Western ethnic minorities, liberal beliefs were associated with less informal help-seeking behaviors, perhaps due to fear of judgment from their community based on their attitudes towards sexuality [28].

There are a variety of barriers to children’s help-seeking behaviors in the context of CSA. These include a lack of education and socio-economic barriers, such as a lack of resources and economic disempowerment. Additionally, psychological barriers such as fear of ostracism from the community, shame, and lack of belief [24] have been found to impact whether children seek help. Socio-cultural norms regarding patriarchy, sexual taboos, blame, and modesty may also stand as impediments to children’s help-seeking behaviors [28]. Furthermore, gender may be a barrier to help-seeking behaviors in relation to CSA, both for girls and boys. For example, power inequalities regarding gender may hinder girls and women from seeking help due to disempowerment and fear of not being believed due to society’s tendency to dismiss girls’ and women’s voices [24]. Boys and men also face barriers to help-seeking due to their gender, such as feelings of embarrassment, a lack of communication skills needed to seek help, and lower levels of accessing professional and social support compared to girls and women [27].

### 1.2. Responses of Educators to CSA Survivors’ Help-Seeking Behaviors

CSA survivors have identified educators as taking on numerous roles, ranging vastly from the person who committed the CSA, to a bystander, supporter, or that of a savior [29,30]. Similarly, educators have been found to play crucial, although complex, roles in CSA prevention, detection, intervention, and coping [5,30,31,32]. Consequently, educators are often disclosure recipients as they are perceived by children to be trusted figures [3,33]. However, a school’s culture greatly impacts educators’ responses to students’ CSA disclosures and help-seeking. Several factors influence how educators handle disclosure, including training and familiarity regarding CSA identification, treatment and reporting policies [32,34,35], and their perceptions of professional responsibilities [22,36,37]. For example, some principals have been found to take an active leadership role, while others take a passive avoidance approach [38], and some act in solitude, while others share the responsibility with colleagues [30]. Among teachers, perceptions of their power, control, responsibility, and ability to act in regard to CSA cases have been found to diverge based on contextual factors, such as religion and socio-cultural and socio-political positions [7,39]. Previous positive or negative experiences may also deter or encourage educators to report suspected abuse [40].

A crucial intervention in this context is the improvement of educators’ early reporting of suspected CSA, as this has been shown to decrease the short- and long-term consequences [32]. For example, CSA training programs for educators have been associated with increased self-confidence when identifying CSA [35] and higher disclosure rates [41]. Despite such achievements noted in these and other studies [31,42,43], it has been found that educators generally lack the necessary training to address CSA effectively [42]. Nevertheless, the literature has highlighted the importance of the responses to CSA by key adult figures in children’s lives, including education professionals. Thus, training programs for educators should promote an institutional and educational culture of awareness and empathy in response to help-seeking and CSA disclosure to minimize CSA perpetration and support survivors [44].

### 1.3. CSA Survivors’ Perceptions of Social Responses to Their Help-Seeking Behaviors

The social responses to CSA survivors’ disclosures and help-seeking behaviors can play a crucial role in providing effective treatment and support for survivors [45,46]. Positive responses to CSA disclosure include the recipient being supportive and calm to encourage the survivors to feel safe and accepted. In contrast, survivors have identified negative responses, including insensitive, minimizing, and dismissive reactions, as well as over-aggressive reactions toward the person who committed the CSA [47]. Research has found that positive responses can be protective factors for survivors. Conversely, responses perceived as hurtful by the survivors may lead to further psychological and physical harm and exacerbate the levels of post-traumatic stress disorder and anxiety [48]. Although perceptions of social responses have been found to vary between boys and girls who have experienced CSA, negative social responses may hinder recovery for all survivors [46].

### 1.4. The Israeli Educational System and CSA

In Israel, educational institutions are tasked with implementing specific guidelines regarding CSA interventions. This requires conducting annual workshops to review school procedures, while ensuring that teachers have the necessary skills, knowledge, and protocols to conduct CSA reporting as directed by the Director General’s Circular [49] of the Israeli Ministry of Education. Nevertheless, Israeli educators have been found to have limited knowledge regarding the prevalence of CSA among their students [50], which is estimated to be approximately 19%, according to self-reports and other data [36,51]. While educators are required to report any circumstance of suspected abuse of a minor [52], the main figures involved in interventions after a CSA disclosure are typically those working in child protection and welfare services, investigators, police officers, legal experts, and medical professionals [53,54]. Consequently, educators are often excluded from CSA interventions, despite being the disclosure recipients [6].

In the Israeli context, studies conducted with CSA survivors have primarily referred to the education system as the arena in which the abuse took place, perpetrated by school employees [55] and peers [56]. A report by ARCCI found that 5% of reported CSA cases were perpetrated by educational figures [57]. A later report by the Israeli National Council for the Child (NCC) determined similar rates in 2019, with 2.6% of child survivors reporting to investigators that the person who committed the CSA was an educational figure [58]. Furthermore, the Israeli Child Online Protection Bureau, which receives thousands of inquiries concerning sexual offenses toward children yearly, found that 10% of cases were perpetrated by educators [59], while other research has found that 46% of cases reported to rape crisis centers involved student peers [60]. Regarding help-seeking patterns of sexually abused students in Israel, a study conducted with Israeli adolescents revealed that over 60% of those who had experienced CSA tried to seek help from a school official [61]. In another study, one survivor’s reason for not reporting was a lack of attention to student complaints and children’s expulsion from the school following complaints [62].

### 1.5. Context of the Current Study

From 2020 to 2021, the Israeli Independent Public Inquiry into CSA collected 367 written accounts from CSA survivors regarding their experiences. The Inquiry members are recognized experts and practitioners in child maltreatment, law, social work, research, and social activism. The Inquiry aims to inform policy changes and develop improved services for all children. In addition, the Inquiry’s work originates from the awareness that CSA survivors have valuable and unique experiential knowledge. Accordingly, it is imperative that all CSA prevention and intervention approaches incorporate their knowledge and perspectives. To fulfill this aim, the Inquiry asked adult CSA survivors to share their narratives regarding their experiences of CSA to inform future policies and recommendations for research.

## 2. Methods

Given the limited literature and the complexity of the phenomenon, a qualitative approach was chosen for the present study. This approach allowed for a multifaceted exploration of the meanings ascribed by CSA survivors to the educational system and educators, based on their own perspectives and experiences [63]. A descriptive phenomenological perspective guided the study, as this qualitative methodology is suited to reveal the perceptions and meanings the survivors attributed to their educators and educational system’s responses to their manifestation of CSA [64]. This approach enabled the researchers to capture the lived experiences of the CSA survivors. 

### 2.1. Research Procedures

The Inquiry put out a call for individuals over the age of 18 to provide testimony and share their experiences of CSA that had occurred from the ages of 0 to 18. To ensure the inclusion of a diverse group of CSA survivors, the call was published in five languages. It was promoted through multiple channels, including online social networks and media (e.g., radio and newspapers), and endorsed by social influencers and public figures. The data were collected using two platforms. Thus, the participants could choose to complete an online questionnaire with the option to submit a written or audio account of their experiences or have a face-to-face meeting with an Inquiry team member. 

In each platform, the following procedure was carried out: (1) Demographic information was requested, including age, gender, and education level. (2) This was followed by questions related to the abuse, such as: (a) the age when the abuse began; (b) the identity of the person who committed the CSA; and (c) the duration of the abuse (e.g., was the abuse a one-time occurrence or prolonged). (3) Next, open-ended questions were asked that related to the abuse (e.g., Please tell the story of the abuse in your own words. Was the abuse revealed during childhood, and if so, please share with us by whom? When? Or to whom? What were the reactions—if any—to the disclosure of the CSA?) and receiving help (e.g., Please share with us whether you received help as a child, for example, personal, familial, social welfare, legal, or other). (4) Finally, the participants were invited to share any messages they had for the public institutions that address or encounter CSA, including the educational system, in their testimonies.

### 2.2. Study Sample

The current study drew data from a total of 367 CSA survivors’ testimonies submitted to the online self-reporting questionnaire platform. Keywords referring to the education system were searched for among the testimonies to identify those relevant to the study. The keywords most commonly used by the participants were education (*n* = 107), teacher (*n* = 91), school (*n* = 76), and class (*n* = 71). After removing the narratives that did not mention the education system, the final sample comprised 61 testimonies. Please refer to Table 1 for the participants socio-demographic characteristics. Women constituted 77% of the participants, and 23% were men, with ages ranging from 17 to 65. Of the participants, 79% reported experiencing ongoing abuse, and 21% reported a one-time event, with the age of abuse onset ranging from 3 to 17. The CSA experiences often involved more than one person who committed the CSA. The people who committed the CSA comprised multiple identities, including individuals directly (e.g., teacher, driver, and peers) or indirectly (e.g., parent of a classmate and teacher’s spouse) involved in the education system as well as family members, neighbors, other acquaintances, or strangers. Testimonies were selected and included in the sample if they referred to at least one experience involving the education system.

### 2.3. Qualitative Thematic Analysis

Braun and Clarke’s [65] thematic method was implemented to identify, analyze, and organize the themes that arose from the testimonies. Specifically, the authors employed reflexive thematic analysis, which prioritizes the researchers’ reflexivity throughout the stages of the analysis, involving a deep introspection of their perspectives, interpretations, and biases [66,67]. During the analysis process, the research team was engaged in reflexivity in several ways, including training the team members regarding their role in the research; maintaining reflective journals to record thoughts, biases, and assumptions; prompting team members to critically examine their backgrounds and experiences that could affect data interpretations; and holding regular meetings to reflect on the emerging themes and interpretations.

This study is rooted in the constructivist epistemological framework, aligning with many qualitative studies [68], which emphasize that people construct their own understanding of the world. As such, it values the exploration of individuals’ complex and subjective experiences and views meanings as products of social and historical contexts, encouraging the inductive development of theories from observed data. According to this approach, the researcher’s role is also recognized in data interpretation and, therefore, the researchers maintained reflexivity in all research stages. 

To gain familiarity with the data set, the authors read each testimony independently. Next, the units of meaning were identified, coded, and categorized according to the content. The established categories were further discussed among the authors in peer debriefing meetings, and initial themes were organized accordingly. Each theme was then reviewed, named, and defined. The theme interpretations were compared to the raw data to confirm that the representations of the participants’ experiences were accurate.

Lincoln and Guba [64] outline the four criteria of transferability, credibility, confirmability, and dependability to achieve trustworthiness in thematic analyses. To adhere to these criteria, and in consultation with Nowell et al. [69], the following steps were taken. To achieve credibility, the participants were provided with a platform to express themselves in their own words during the testimony. Each author conducted an independent analysis to ensure the participants’ perspectives and experiences were conveyed authentically. This was followed by discussions to reach a consensus on the findings and their interpretations. Transferability was obtained by providing detailed descriptions of the research process. Dependability was reached through the documentation and audit trails of the decision-making processes. In addition, peer debriefing was conducted to ensure accuracy and counteract the authors’ subjectivity during the analysis. Overall, the attainment of confirmability can be attributed to the preceding criteria, which serve as evidence of the authors’ meticulous approach to accurately portraying the participants’ viewpoints and experiences. 

### 2.4. Ethical Considerations

This study was conducted in accordance with the Declaration of Helsinki and approved by the ethics committee of Tel Aviv University, approve number: 0001408-2 (8 June 2021). The authors also endeavored to adhere to the highest ethical standards. Informed consent was a prerequisite for participation, and the participants’ confidentiality was assured. During the Inquiry’s data gathering, the participants were informed during the Inquiry that their participation was voluntary and that they could refuse to answer any of the questions in the study. Security protocols were used for the online platform to ensure confidentiality, and only the research team had access to the submitted testimonies. Additionally, all participants were provided with a list of free counseling services and the Inquiry’s email to contact the research team if they needed support or further information.

## 3. Results

**Figure 1:**
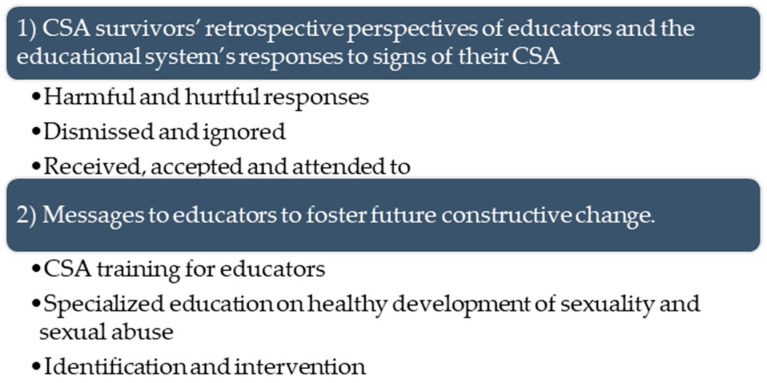
Themes generated from the participants’ CSA narratives.

The findings revealed that the majority of the participants attributed great meaning to the role of educators and the education system in their CSA experience. Their narratives expressed both the vulnerability and important roles embedded within the educational setting. They described the educational system as an arena with a risk for abuse, as well as a space for disclosure and intervention. Educational spaces were often the place where they expressed signs of their abuse or disclosed it. Thus, the educational system and educators played an essential part in their CSA narratives. Two interrelated themes were generated from the analysis and are presented in Figure 1: (1) CSA survivors’ retrospective perspectives of educators and the educational system’s responses to signs of their CSA and (2) messages to educators to foster future constructive change.

### 3.1. “I Showed Many Signs, If Only There Was Someone Who Could Read Them”: CSA Survivors’ Retrospective Perspectives of Educators and the Educational System’s Responses to Signs of Their CSA

The participants reported expressing that they were experiencing CSA in various ways. At times, these expressions were subtle and implicit (e.g., psychosomatic reactions, behavioral arousal, or avoidance), while others were outwards and direct. Responses to the CSA survivors’ differing manifestations of displaying or disclosing the abuse were described as ranging from abusive to life-saving and included (1) being harmful and hurtful; (2) being dismissed and ignored; and finally, (3) being received, accepted, and attended to by the educators.

#### 3.1.1. Harmful and Hurtful Responses

On the extreme end of the spectrum, the participants described educators’ responses of blaming the survivor or taking seemingly supportive actions that the survivors experienced as careless and harmful. In some cases, when the survivors actively sought help, even with the support of their parents, they were met with acts of blaming and shaming by the educators. One participant described the following:

When the principal learned of the abuse by one of the school parents, from the parents of a student who was sexually harassed, he claimed to my mother that he could not file a complaint and did not report it. I was then banned for talking about the abuse to the other girls in the class. […] [A] few days after, there were police investigators at the school (after I complained on my mother’s initiative), a talk was given to the students about modesty, hinting that if they remained modest, things would not happen to them.

In other cases, interventions from the educational system were experienced as indirectly harmful to the survivor. One participant described how she and her sister were raped by her father and brothers, sometimes in front of their mother, who demanded that they not disclose the abuse. Although they did not actively disclose the rapes, their call for help was revealed in their outward behavior. She described the reaction of the educational staff regarding the abuse:

At school, they noticed a change in our behavior. When they tried to talk to us about whether something was happening at home, sometimes we were silent, and sometimes we said nothing was happening […] because we were afraid […]. The mistake they made was that they would invite our parents to a conversation […] they asked, what is the girl going through?! And my parents played righteous, loving and caring...?? At home we received a reprimand, anger and a punishment that we dared to hint that something was not well […] Instead of the school being a lifesaver, it was just the opposite.

Although the sisters’ behavior raised concerns among the education professionals about their environment at home, the intervention involved the abusive parents. This led to further danger and harm for the victims and may have ended their hope for salvation. Thus, while the school did not ignore the sisters’ behaviors, its reaction led them to be at further risk.

#### 3.1.2. Dismissed and Ignored

The majority of the participants described school environments that were not attuned to indications of CSA and, consequently, were unable to recognize the behavioral patterns indicating abuse, such as nonverbal attempts to make their distress known. The participants hoped that the adults in their educational environments would identify the signs and were disappointed with the system when it failed to do so:

As a teenager, when I was already suicidal, I tried to get help from a school counselor […] I spoke in a very general and vague way because it was a first meeting, and I did not trust the counselor yet […] She told me, “Everything is fine with you. Children come to me from your class who have terrible things happening at home; believe me, this is not what you’d call a problem.” She patted me on the shoulder, accompanied me back to class, and I realized I had no one to turn to.

After only one meeting, initiated by the CSA survivor, she was dismissed, ignored, and patronized for her request for help. Thus, she immediately lost trust that the educational figures could assist her.

Another participant described displaying various signs of extreme distress in school, which were not addressed despite the authority figures being aware of her state and behavior:

I was the girl with the dark circles under her eyes […] The girl who was always looking to put her head down for another hour of sleep, the girl who did not eat at all and other days did not stop eating. The girl who could not stand noise, the girl who did not stop apologizing, the girl with recurrent urinary infections […] the girl with covert suicide attempts […] The girl with the pains that make everyone roll their eyes and remind her that children do not have joint pains or headaches. The girl who later was promiscuous with the boys in the class, and in retrospect, everyone, including the social workers, including the teachers, and including the parents, knew. Me? I never received help or support.

For many of the participants, their manifestations of needing attention were not only ignored but also reframed to center them as the cause of the problem. Therefore, external signs indicating high levels of distress were instead framed to focus on the child’s poor behavior:

In middle school, I did not come to school about two-thirds of the time I was supposed to, and other than stating it as a behavior problem on the report card, nothing, no reaction. In high school, I tried to commit suicide–nothing.

Regardless of the gravity of the signs this CSA survivor displayed, no one reacted. Their chronic absences were not recognized as worthwhile to delve into and understand. Even after their suicide attempt, the participant still felt invisible.

Even when the participants’ calls for help were blatant, some school officials’ responses were neglectful and expressed ignorance, as represented in the following quote:

[T]he school nurse came into the classroom and said we were going to talk about sex education once a week […] We were asked to write on anonymous pages the topics we’d like to deal with. The nurse did not read my note. I wrote there “sexual abuse.”

The survivors were hurt by the various systems’ failures to identify and respond to their distress. Furthermore, instead of receiving help, many of the participants were often treated as problematic and subjected to monitoring and supervision because of their behavior. As one participant vividly described,

My sex education teacher was the mother of the one who abused me. I had outbursts in class, especially in her classes, and kept getting punished and reprimanded. They’d call my mom endlessly and threaten that if my behavior did not improve, they’d dismiss me from the school […] No one really bothered to think and check why I was behaving like that. A child does not just act like that.

In some cases, the educator’s ignorance was manifested in avoiding taking basic actions to protect the survivors. One participant explained:

The same friend reported [the abuse] to the principal […] but he did not remove the offender from the yeshiva [religious school for boys]. The guy kept trying to abuse me in the mikveh [ritual bathing area] or wherever he found me alone. After a while, the guy was thrown out of the yeshiva after more guys reported that he’d abused them.

In this important example, the school’s failure to act to remove the person who committed the CSA promoted further abuse attempts.

#### 3.1.3. Received, Accepted and Attended To

The education system and educators also held important positive roles for the participants as they were recipients of the CSA disclosures and the first to intervene. A participant described the importance of the school counselor in the post-disclosure intervention:

And she [the counselor] directed me to go for treatment at [gives the names of two treatment centers specializing in sexual abuse]. I was with her for three years and she rehabilitated me, got my head on straight, to this day, I appreciate her.

Another participant described her high school educator as a lifesaving figure:

At the age of 17, I ran away from home with the help of my school teacher […] I said my dad touched me and she helped me right away […] I did not agree to contact the police, so she simply arranged for me to have an informal foster family, without contacting the welfare authorities. A month after running away, I had appendicitis, so they brought my dad to sign a consent form for surgery. By then I had already lodged a complaint with the police and […] welfare said that I could stay with the foster family.

Here, significant support for the survivor was described, which included careful attentiveness to her specific requests, while maintaining her sense of control and ability to choose in an uncertain situation.

Thus, educators’ responses to CSA survivors’ differing calls for help were described as ranging from helpful and lifesaving to abusive, with the middle ground characterized by ignorance and a lack of attentiveness that did not aid in ending the abuse. This variety of post-disclosure encounters defined the participants’ perceptions of the role of the educational system and educators in their abuse stories, alongside cases where the school was directly involved in the abuse or disregarded signs of the participants’ abuse. Based on these experiences, the participants were asked to convey essential messages they wanted to give to educators, which are described in the following section.

### 3.2. “Pay Attention!!!”: Messages to the Education System to Foster Future Constructive Change

The participants sought to convey messages to educators. While in some of the CSA cases, educators served as saviors, in many others, the participants expressed criticism and disappointment. In their messages, they noted their expectations that educators would have a central role in the CSA prevention, detection, and intervention processes. They most strongly sought to convey the need for educators to: (1) receive professional training, (2) provide beneficial sexual education to their students, and (3) be able to identify and respond to CSA manifestations and/or disclosure in valuable ways.

#### 3.2.1. CSA Training for Educators

The participants described the need for teacher training to include instruction on sexuality and sexual abuse to ensure they have a level of expertise when they step into their positions as educators. The participants repeatedly used the word “training” in many of the testimonies in strong, even passionate, terms. One participant said: “Currently, the teachers do not receive any training for this difficult situation in their classrooms.” Another participant stated, “Every teacher must take at least one course on sexual abuse for his or her degree—identifying children at risk and promote more discourse around this.” Another participant asserted,

I think it is very important that knowledge [on this topic] is present in the training of educators […] to be aware of such situations, to know the statistics, to keep an eye on the relevant factors and to have procedures for dealing with situations that identify abuse, grounded in protocol. Sensitivity in this area is very important, ability to identify subtle symptoms like self-hatred and recognize symptoms of various kinds of self-harm.

The notion of training comprised two main areas in which the participants believed teachers required expertise: the ability to identify abused children and the ability to teach children about healthy sexuality and sexual abuse.

#### 3.2.2. Specialized Education on Healthy Development of Sexuality and Sexual Abuse

The participants shared the belief that the school system should be involved in educating children on concepts related to sexuality in general, not only sexual abuse prevention. They felt that schools’ handling of this issue was insufficient:

Sex education is one of the most useless lessons in our [education] system. Every year they would show us the same cartoon, and what I remember from it is a boy jumping into a pool with an erection. Talk to kids about sexuality! There are already programs about healthy sexuality, they just need to be promoted and introduced in every school.

Another participant stated,

First of all, there should be real sex education in every school, and not two or three lessons on the reproductive system and some video animation about an egg and sperm. Something that will last for a whole year, a lesson that teaches communication and listening, consent, boundaries, mutual respect, and empathy and sensitivity.

Some participants specifically addressed sex education in religious or traditional communities, including Arab, Jewish ultra-Orthodox, and Jewish national religious societies:

In my opinion, the scope of the phenomenon is wider than reported, especially in closed communities. We must find a way to enter ultra-Orthodox communities and the like, this terrible feeling that I have no one to talk to at all, that the teacher will judge me or that there is no counselor at all in the school.

The participant’s following message specifically related to the Arab education system:

The Arab [education] sector lacks a lot of knowledge and discernment in situations in which the child is in danger. Because society decides to remain silent instead of creating a public disgrace and because it is too sensitive an issue, sex education must be mandatory and delivered to children by competent bodies from an early age.

The participants also expressed their concern about the Jewish ultra-Orthodox education system:

One should pay attention when a third-grader sits under a table all day, that it’s not nothing. In secular and even national religious schools, there are sex education classes […] For the ultra-Orthodox, there’s no way to go into the issue of sexual abuse at all, and yet one can discuss putting up personal boundaries without going into detail, what’s allowed and forbidden and that you’re allowed to say no to an adult.

#### 3.2.3. Identification and Intervention

In their messages to educators, many participants sought to convey the necessity of educators acquiring expertise in identifying CSA based on children’s behavior. Educators were framed as central figures for disclosure, even before parental figures. Yet, most participants described how, in practice, the educators in their lives found it difficult to appropriately interpret their behavior and attempts to express their distress. This may explain the participants’ urgency in asserting the need for training to create the necessary environment for disclosure to occur. One participant shared their frustration with the misinterpretation of children’s nonverbal communication:

I was in boarding school and I would hit everyone. […] I would not talk, I would cut myself. Today I know I hit not because I wanted to hit, but because no one heard my cries. A child does not want to be problematic. A child who comes and hits people in school, he does not just hit. Maybe they beat him? Maybe hurt him? Maybe it’s something he learns at home? A child does not just go wild and become an animal. Even a dog does not just behave the way he does, only if he’s harmed. You need to pay attention.

Another participant asked educators to pay attention to the behavior of introverted children whose distress is quietly expressed:

I think we need to focus on the quiet and introverted kids like I was, check on them in particular, what’s going on there? Do not focus only on children who make noise and disturb, pay attention to those who go unnoticed.

Moreover, the survivors requested that educators go beyond simply noticing overt and hidden signs of potential abuse. They told educators to proactively clarify their suspicions and ask explicitly and clearly if abuse has occurred. One of the participants implored,

Pay attention! Pay attention! Pay attention! Pay attention!!! I would come to school with cuts on my hands, and no one asked anything. I would cry in class, skip out on everything, depressed about life—and it did not interest anyone. If someone had asked, maybe, “What’s going on?” things would be different.

The participants asked educators to understand that most of the time, their students will not directly tell them they have been abused. Instead, they will manifest their pain, whether overtly in extroverted behaviors or covertly in introverted behaviors. 

These messages reflect the centrality the participants gave to the education system in their abuse stories. First, this abuse may occur within or around the educational setting and be perpetrated by adult figures or peers present in that space. Yet, educational settings and figures are also key disclosure recipients, with most participants describing their destructive behavior at school as a nonverbal attempt to disclose the abuse. These behaviors were often misinterpreted and seen as deviant. Moreover, after disclosure, the reactions of the education system were perceived as ranging from life-saving to abusive by denying, blaming, or intervening in an inappropriate way.

The participants attributed great importance to educators in the context of children’s sexual development. They advocated for better educator training in this matter as well as for appropriate sex education to be conducted in schools. Significantly, the participants hoped that educators would learn to recognize signs of distress in their students and be aware that inappropriate behavior should prompt teachers to search for an explanation. Educators were also asked to learn to develop skills to respond in beneficial, sensitive ways to support the treatment and recovery of CSA survivors.

## 4. Discussion

This study aimed to explore the perspectives of CSA survivors regarding educators and the educational system. The extensive literature addresses how educational institutions should function as places where children feel socially, emotionally, intellectually, and physically safe [70,71]. Educational settings have the potential to not only foster children’s development but also provide a sense of security and thus are vital to abuse disclosure. However, in the current findings, they were equally described as spaces of vulnerability, violence, and danger [13,72]. Moreover, the findings indicated the dialectical nature of the educational environment. While schools can function as sanctuaries where, children find solace and support, they may also be environments where, children experience abuse or harmful responses to their distress. Therefore, the current study highlights the critical importance of providing educators with the necessary training and empowerment to identify early, subtle, and behavioral indications of abuse or distress. 

Raising the voices of CSA survivors is an essential mechanism of transitional justice [45], the process by which a society “attempts to come to terms with a legacy of large-scale past abuses” ([73], p. 4). Advancing and listening to the voices of survivors is crucial in the quest for justice and allows for learning from the past and generating new knowledge and recommendations [45]. Moreover, listening to survivors’ voices and allowing their perspectives to inform practice, policy, and research is critical for the effective support and treatment of CSA survivors. Society’s ability to listen to and protect survivors’ voices, perspectives, and disclosure is fundamental to promoting children’s sense of safety in disclosing and seeking help, and thus critical for the healing process and justice for CSA survivors [74].

Highly notable findings were related to the significance of educational institutions and personnel in identifying children’s help-seeking processes. Help-seeking processes are defined as searching for and using formal or informal assistance in addressing concerns or challenges [24]. Specifically, the reactions of educators and the educational system to both overt and covert help-seeking manifestations in CSA cases appear to have been largely overlooked in previous research [30]. The present study sought to fill this gap and illuminate the education system’s dual role as both a barrier and facilitator following the help-seeking actions of CSA survivors. 

Help-seeking involves non-linear processes rather than one-time decisions and actions [75]. This characteristic pattern was clearly expressed in the survivors’ testimonies in the current study. Thus, the survivors pointed to many indirect, inconsistent, and repeated CSA disclosure attempts. In accordance with the previous literature that has described models of CSA disclosure, the educators and educational system in the current study met the abused children at three crucial points: (1) when they were withholding the secret; (2) when they felt the “need to tell,” and; (3) after they disclosed the abusive event or events [76,77]. In the first stage, the internal desire to tell was sometimes expressed via changes in behavior or psychosomatic physical signs, a process known as the “pressure cooker effect” [77,78]. Consistent with this pattern, the participants described their longing for the educational staff to recognize their distress and desperate willingness to be asked direct questions about the CSA.

The most commonly identified factor promoting disclosure is being asked about the abuse by trusted individuals [79]. The participants identified some educators as facilitating the “opportunity to tell” ([78] p. 210) by giving full confidence to the child and their abuse story. Unfortunately, numerous testimonies suggested that many educators impeded the CSA help-seeking manifestations due to their failure to accurately interpret the participant’s troubling behavior [76]. In cases where disclosure did occur, the observed responses ranged from supportive to complacent attitudes toward reporting policies to disregard, mistrust of the disclosure, and a lack of involvement [13,76]. In light of this, the survivors in the present study called on educators to take proactive steps to advance identification, disclosure, and intervention mechanisms in CSA situations.

The findings indicate that the CSA survivors held primarily negative perceptions of the support they received from educators within the educational system. Even so, there were clear parallel lines between what the survivors who participated in the study called for and what the literature has indicated as being highly beneficial to teachers in the context of coping with CSA. The survivors called for training, sexual education, and the provision of CSA knowledge to identify and intervene. Interestingly, similar calls have been found in studies on the perspectives of educators coping with the CSA of their students, including the importance of more training for educational staff, student sexual education, and knowledge access for the school community [80]. Teachers have also reported challenges due to the expectations of their personal resources, such as time and mental health, in addressing CSA, while encountering a lack of support from the educational system, the silencing of their needs, and the ignoring of their voices [7,22]. Thus, all the social actors involved in these circumstances are looking for help and support, with clear parallel help-seeking of both the survivors and the teachers, with the teachers’ experiences as an additional external manifestation of the survivors’ experiences.

In this study, while the survivors prominently voiced dissatisfaction regarding the responses and actions of educators in recognizing and addressing CSA, the findings also illuminated its potential for becoming an arena for transitional justice. Thus, a supportive, protective, and responsive reaction to survivors’ CSA disclosures and help-seeking behavior was found to be crucial for effective intervention and the survivors’ wellbeing. Therefore, implementing justice-sensitive education, which promotes transitional justice and transformative structural, institutional, and curriculum development [81], could be highly valuable. Justice-sensitive education has the ability to address CSA and its society-wide implications and trauma and includes justice-informed training for teachers and the overall school community. It acknowledges and addresses the abuse experienced by CSA survivors, not only from the people who committed the CSA but also from the society at large that silences and ignores their voices and needs. Justice-sensitive education searches for the truth and raises awareness regarding CSA by voicing the perspectives of survivors and looks to a better future by seeking interventions and treatments based on survivors’ perspectives.

Simultaneously, transitional justice also calls for listening to the voices of educators who also experience the traumatizing effects of CSA [82]. Teachers face high personal costs, fear, loneliness, and an ever-present conflict between their professional and personal identities when facing the CSA of their students [30]. Schools, as contexts for transitional justice, are also crucial spaces for empowering and supporting educators as agents of change, which is instrumental in their frontline role in empowering survivors and promoting CSA intervention, prevention, and disclosure.

### 4.1. Limitations

In addition to the current study’s findings, the following limitations should be noted. The testimonies in this study were based on the participants’ retrospective interpretations. Therefore, the data do not necessarily reflect the participants’ experiences at the time of the abuse. However, in qualitative research, the trustworthiness of participants’ recollections is secondary to their associated impressions, attributed meanings, and personal perceptions [83]. It is also important to acknowledge that this study was based on written testimonies and not in-depth interviews. Some of the participants’ descriptions were concise, which limited the rich descriptions of their perceptions, attitudes, and feelings.

Furthermore, the study did not account for different educational institutions (e.g., boarding school, residential care, elementary or high school, and yeshiva), as this was not directly stated in the majority of the testimonies. Future research should address the aforementioned multiple contexts in light of the differences in organizational cultures and CSA risk. Similarly, although the testimonies referred to various professionals (e.g., principals, teachers, and counselors), this study did not distinguish between these groups. The roles of educational professionals should be considered in future research as they hold different responsibilities and tools for CSA interventions. 

In addition, although it was beyond the scope of the current study, the participants experienced various forms of CSA (e.g., within and outside the family, by peers, inside and outside the educational institution), which may have shaped their perceptions of the educational system. Follow-up studies should address the impact of different types of CSA. Additionally, each participant’s specific characteristics were not available unless they provided them during their testimony. Furthermore, the analysis included culturally heterogeneous testimonies, including references to mainstream Israeli society, Arab society, and religious and ultra-Orthodox Jewish societies. Given the dramatic variability regarding patriarchal structures, cultural and religious concepts, and attitudes toward CSA reporting and intervention, further research should examine the topic from a culturally informed perspective and conduct comparisons between communities.

Finally, the current study did not consider the power dynamics between the participants when they were students and their educators. As students and educators are not equal partners, the influence of this power differential on students’ experiences of CSA in educational contexts is an important area for future examination. Furthermore, given the time since the CSA incidents and when the testimonies were collected, the findings may not reflect advances in social recognition and school policies in the intervening decades.

### 4.2. Implications

In Israel, there is no legislation requiring dedicated training for professionals, such as educators, whose work implicates them in CSA intervention processes; likewise, in teaching academies, there is no obligation to offer a specific course on the subject [84]. The concerns voiced by the CSA survivors in this study point to the need to establish a dedicated training system focused on educating educators to recognize non-verbal signs of CSA, including silent, help-seeking behaviors [77]. The survivors indicated that being asked directly about their external symptoms of distress can alleviate the difficulty of disclosing the abuse [79]. Additionally, institutional policies to combat grooming are essential, including a clearly delineated code for what constitutes improper behavior toward children and an approved procedure for responding to such warning signs [85].

The survivors also highlighted the key role of the education system in promoting education regarding healthy sexuality and sexual abuse. In Israel, the Ministry of Education conducts a compulsory program (called “Life Skills”) in elementary and middle schools that deals with sex education and sexual violence, among other topics [86]. However, in line with previous studies, the participants indicated that the sex education they received was brief and inadequate. Consequently, they suggested that sex education should move beyond biological functions, strengthen children’s communication skills, and be taught in a safe class atmosphere based on dialogical and reflective approaches [87].

The testimonies referred specifically to the lack of sex education in Israel among closed collectivist communities [88]. Sex education programs are frowned upon in the Arab community [89], and in schools where they do exist, the discussion of intimate body parts may not be allowed [90]. In the Jewish ultra-Orthodox context, despite an impressive increase in programs addressing CSA in the last decade [91], such programs do not yet include content addressing sexuality. Furthermore, the presentation of topics related to sex and sexuality must be carefully worded and culturally modified [92]. Nonetheless, the participants viewed discourse on sexuality as a necessary condition for promoting child protection and, therefore, called for a paradigm shift in these communities.

At the global level, the implications of the current findings promote moving beyond individual-level interventions to focus on improving institutional climates and organizational cultures as they relate to CSA. For this purpose, it is necessary to promote a cohesive-holistic approach to CSA interventions. Such interventions must involve all members of the educational community, including children, education personnel, administrators, and parents, as well as the wider educational system. In all socio-cultural contexts, educators require ongoing training to recognize signs of abuse and respond supportively to disclosures of CSA in cooperation with the relevant welfare and medical professionals as well as law enforcement. Moreover, age-appropriate sexuality education is vital, as is the establishment of safe spaces within schools. Finally, effective prevention programs should prioritize children’s rights and ensure their factual understanding of CSA.

In addition, this research demonstrates how the educational system and its personnel have a significant and central role in identification and intervention. CSA survivors see the educational system and educators as meaningful and important figures in their experience of their CSA. Therefore, educators must be full partners in professional teams dealing with the sexual abuse of children and should be involved and updated when making decisions. This may empower them, enrich their knowledge and training, and help increase their professional support for their students.

## Figures and Tables

**Table 1:** Socio-Demographic Characteristics (*n* = 61).

Variable	Total	
	N	%
**Gender of victim**		
Male	14	23%
Female	47	77%
**Age today**		
17–32	22	36%
33–48	27	44%
49–64	11	18%
65+	1	2%
**Age at the time the abuse occurred**		
0–5	24	39%
6–11	23	38%
12–17	14	23%
Perpetrator identity *		
Educator	16	17%
School staff	6	6%
Family member	18	19%
Parent	11	11%
Religious leader	3	3%
Peer	8	8%
Acquaintance or known individual	23	24%
Medical staff	2	2%
Intimate partner	1	1%
Stranger	3	3%
Unknown	5	5%
CSA disclosure recipient identity *		
Educator	6	9%
Relative	5	8%
Parent	11	17%
Peer	4	6%
Mental health professional	4	6%
Undisclosed	17	26%
Disclosed only in adulthood	8	12%
Unknown	10	15%
Place of abuse *		
At home	14	21%
Educational setting	14	21%
Religious educational setting	6	9%
Outside	6	9%
At the perpetrator’s home	11	16%
Unknown	16	24%
**Duration of abuse**		
Ongoing abuse	48	79%
One-time	13	21%

* Frequencies of perpetrator identity, recipient of CSA disclosure, and place of abuse may appear higher than participant frequency due to participants reporting multiple abuse incidents.

## Data Availability

The data are not available due to confidentiality and ethical restrictions.
